# Implementation of basic life support education for the lay public in China: barriers, enablers, and possible solutions

**DOI:** 10.3389/fpubh.2024.1390819

**Published:** 2024-06-27

**Authors:** Xuejie Dong, Lin Zhang, Zongbin Wang, Zhi-jie Zheng

**Affiliations:** ^1^Department of Global Health, School of Public Health, Peking University, Beijing, China; ^2^School of Public Health, Shanghai Jiao Tong University, Shanghai, China

**Keywords:** basic life support (BLS), lay public education, emergency medical services (EMS), barriers, enablers, implementation, EPIS framework

## Abstract

**Background:**

Education for the lay public in basic life support (BLS) is critical for increasing bystander cardiopulmonary resuscitation (CPR) rates and improving survival from out-of-hospital cardiac arrest (OHCA). Despite years of implementation, the BLS training rate in China has remained modest. The aim of this study was to investigate the factors influencing the implementation of BLS training programs in emergency medical service (EMS) centers in China and to identify specific barriers and enablers.

**Methods:**

Qualitative interviews were conducted with key informants from 40 EMS centers in Chinese cities. The participants included 11 directors/deputy directors, 24 training department leaders, and 5 senior trainers. The interview guide was based on the Exploration, Preparation, Implementation, Sustainment (EPIS) framework. Thematic content analysis was used to identify themes and patterns across the interviews.

**Results:**

We identified 16 factors influencing the implementation of BLS training programs encompassing the outer content, inner context, innovation and bridging factors. Some factors acted as either barriers or enablers at different EPIS stages. The main implementation barriers included limited external leadership, insufficient government investment, low public awareness, a shortage of trainers, an absence of incentives, an absence of authoritative courses and guidelines, a lack of qualification to issue certificates, limited academic involvement, and insufficient publicity. The main enablers were found to be supportive government leaders, strong public demand, adequate resources, program champions, available high-quality courses of high fitness within the local context, the involvement of diverse institutions, and effective publicity and promotion.

**Conclusion:**

Our findings emphasize the diversity of stakeholders, the complexity of implementation, and the need for localization and co-construction when conducting BLS training for lay public in city EMS centers. Improvements can be made at the national level, city level, and EMS institutional level to boost priority and awareness, promote legislation and policies, raise sustainable resources, and enhance the technology of BLS courses.

## 1 Introduction

Out-of-hospital cardiac arrest (OHCA) is a global health issue, with incidence and survival varying substantially by region (1). Survival of patients with OHCA requires a coordinated set of actions, known as the “chain of survival” (2, 3). Early community responses, including immediate recognition and activation of the emergency medical service (EMS) system, early cardiopulmonary resuscitation (CPR) and rapid defibrillation using an automated external defibrillator (AED), have the greatest impact on survival (3). Education for the lay public in basic life support (BLS) targeting community responses has shown great potential to increase bystander CPR rates and survival from OHCA (4–7), and has been emphasized by the formula for survival and international guidelines (2, 8, 9). In the recent years, the concept of “chainmail of survival” was proposed as a modern concept for adaptations of CPR systems. It symbolizes the “protection” from cardiac arrest, advocating for the implementation of layperson education and community first responder systems (10).

Not all communities and individuals have equal access to BLS education or training programs (11). There are large disparities in the coverage of BLS training among regions, and the successful implementation of BLS training programs is mainly reported by high-income countries (12, 13). The Nation of Lifesavers project conducted by British Heart Foundation (UK) (14), the Heart Safe Community project (HSC) in Australia (15), the Home Education and Resuscitation Outcomes Study (HEROS) in South Korea (16), and the Dispatcher-Assisted first Responder (DARE) project in Singapore (17), for example, all contribute to enhancing the national coverage of BLS training and work as model projects around the world. In China, owing to the late start of economic development, BLS training programs for the lay public began in the late 1980s. Despite several years of implementation, the reported training rates have remained below 1% and no updated for a long time (18, 19). EMS centers play a crucial role in providing BLS training to the lay public. As public health institutions directly under the local health commissions, one of the main responsibilities of EMS centers is to popularize knowledge and provide training for their service population (20, 21). With the increasing public demand for EMS care in recent years, a growing number of EMS centers have prioritized BLS education and highlighted development goals. However, the efficiency and effectiveness of EMS training programs are generally unsatisfactory and vary substantially among EMS centers, indicating an urgent need for improvement.

The efficiency and effectiveness of BLS training can be influenced by factors at the national, regional, organizational, and individual levels. Strategies at the national or regional level, such as legislation, top-down buy-in, mandatory requirements for BLS training in schools and when acquiring a driver’s license, have been reported to improve BLS training (4, 22, 23). However, there has been limited exploration of organizational and individual factors and their influence on training implementation. Although experiences of effective training programs have been shared within the Global Resuscitation Alliance (GRA) (24, 25), little is known about the reasons for unsuccessful programs or barriers to avoid during implementation. Current international guidelines for resuscitation education provide detailed recommendations on the content and methods of training, but there is a lack of practical guidance for organizations or cities seeking to conduct BLS training programs, and knowledge on how to facilitate implementation is warranted.

Implementation science models and frameworks provide systematic and integrated guidance to help identify key factors and processes to facilitate implementation (26). The Exploration, Preparation, Implementation, Sustainment (EPIS) framework is a widely used implementation framework developed for public sector service contexts and integrates a multi-level framework across implementation phases (27, 28). It encompasses four well-defined phases that comprehensively outline the implementation process, identification of the outer system and inner organizational contexts, innovation factors that relate to the characteristics of the innovation being implemented, and bridging factors (the interconnectedness and relationships between outer and inner contexts). The EPIS framework also recognizes the fit between the values and needs of implementers and the characteristics of the innovation to be implemented, which is important for sustainment (29).

The aim of this study was to apply the EPIS framework to explore the factors influencing the implementation of BLS training programs in EMS centers in Chinese cities, and to identify specific barriers and enablers throughout the implementation process. Through in-depth qualitative interviews, we gathered insights from key informants from 40 EMS centers across the country, ensuring the representation of regional diversity and universal findings.

## 2 Methods

### 2.1 Design and setting

This qualitative study involved interviews with key informants of BLS training programs for lay public from city EMS centers in China. These key informants were either responsible for, or had the most familiarity with, BLS training programs from each EMS centers. The interviews were conducted from March to October 2023. The study obtained ethical approval from the Joint Research Ethics Board of the Shanghai Jiao Tong University Schools of Public Health and Nursing (SJUPN-HY-202304-5-KS1). Verbal informed consent was obtained from all respondents before the interviews. All participants were informed about the aim of the study and were assured that participation was voluntary, that their responses would be anonymous and that we had no intention of evaluating any specific EMS center.

### 2.2 Sampling and participants

To achieve the maximum representation of the regional diversity in our study sample, we used a purposive recruitment strategy. First, we partnered with the China Resuscitation Alliance (CRA) to invite key informants from EMS centers in all municipalities and provincial capitals in mainland China, with one respondent per center. Second, we employed snowball sampling to include EMS centers in other cities, and stopped recruiting when data saturation was reached, defined as the point at which no new themes or issues emerged.

### 2.3 Data collection and analysis

Qualitative semi-structured interviews were conducted individually, either in-person or through online meetings with respondents by two primary investigators (XD and LZ). The interview guide was informed by the EPIS framework, and included key questions related to the implementation process of BLS training program for the lay public (Table 1). We also asked the respondents about their demographic and work background, including age, major, position, education level and working years. Each interview lasted 1 to 2 h, and all the interviews were audio recorded with consent of the respondents.

**Table 1:** The interview guide informed by epis framework.

EPIS stage	Key questions asked
Exploration	What were the goals and objectives for your EMS center to conduct BLS training program for lay public? What were the barriers and enablers in the exploration stage?
Preparation	How were the training program developed and prepared? What were the barriers and enablers in the preparation stage?
Implementation	Were there any important events prior to the formal program? What impact have them had? What are the barriers and enablers in the implementation stage?
Sustainment	What is the effectiveness and sustainability of the program? What are the barriers and enablers of the sustainability of the program? What are the challenges and possible solutions?

EPIS, exploration, preparation, implementation, sustainment; BLS, basic life support; EMS, emergency medical service.

Thematic content analysis was used to facilitate a deeper understanding of the interviewees’ thoughts. The interviews were transcribed and read repeatedly by investigators to obtain an overall impression. The stepwise development of the coding system was employed starting with the utilization of a codebook developed by investigators based on the interview guides. EPIS constructs were considered but not imposed unless supported by the data. Two investigators (XD and LZ) independently conducted the coding process. They reviewed a subset of the transcribed interviews and individually applied a series of codes to sections of the text to condense data into organized, analyzable units. The codes were then discussed and integrated to develop a codebook. The codebook contained definitions, guidelines for use, and examples of representative quotes appropriate for inclusion in the category. Each interview transcript was then independently coded, and any discrepancies in assignments of codes were resolved through discussions among the research team (XD, LZ and ZJZ). The data coded into each EPIS construct were further reviewed to generate the common assertions regarding barriers and enablers. The MaxQDA 2022 software was used to conduct thematic analysis. The Standards for Reporting Qualitative Research (SRQR) checklist was used as it provides clear standards for reporting qualitative research (30).

## 3 Results

Our final sample included 40 EMS centers across China, encompassing all four operation modes (Supplementary Table S1): independent, prehospital, dependent, and commanding (31, 32). The 40 respondents included 11 directors/deputy directors (D1-D11), 24 training department leaders (L1-L24), and 5 senior trainers (T1-T5). They were all full-time employees of their EMS center during the study period, where the directors/deputy directors held specialized managerial roles, the training department leaders served as both managers and full-time trainers. The 5 senior trainers were either dispatchers or ambulance staffs who worked part-time as trainers, as there was no training department at their EMS centers. The participants had been working at their EMS center for an average of 17.6 ± 7.2 years, and had been involved in BLS training for an average of 10.1 ± 6.2 years (Table 2).

**Table 2:** Main characteristics of key informants.

Characteristics	Key informants (*n* = 40)
Gender = female, n (%)	15 (37.5)
Age (years), mean ± SD	46.9 ± 7.2
Position in EMS center, n (%)	
Director/deputy director	11 (27.5)
Training department leader	24 (60.0)
Senior trainer	5 (12.5)
Years working in EMS center, mean ± SD	17.6 ± 7.2
Years involving in BLS training, mean ± SD	10.1 ± 6.2
Geographic region of China, n (%)	
East	20 (50.0)
Central	10 (25.0)
West	10 (25.0)
City administrative level, n (%)	
Centrally-administered municipality	4 (10.0)
Provincial capital city	27 (67.5)
Prefecture-level city	9 (22.5)
City scale, n (%)	
Megacity (>10 million population)	7 (17.5)
Supercity (5–10 million population)	11 (27.5)
Large city (1–5 million)	16 (40.0)
Small and Middle city (<1 million population)	6 (15.0)
EMS center operation mode	
Independent	1 (2.5)
Prehospital	26 (65.0)
Dispatching	7 (17.5)
Dependent	6 (15.0)

EMS, emergency medical service; BLS, basic life support.

**Figure 1:**
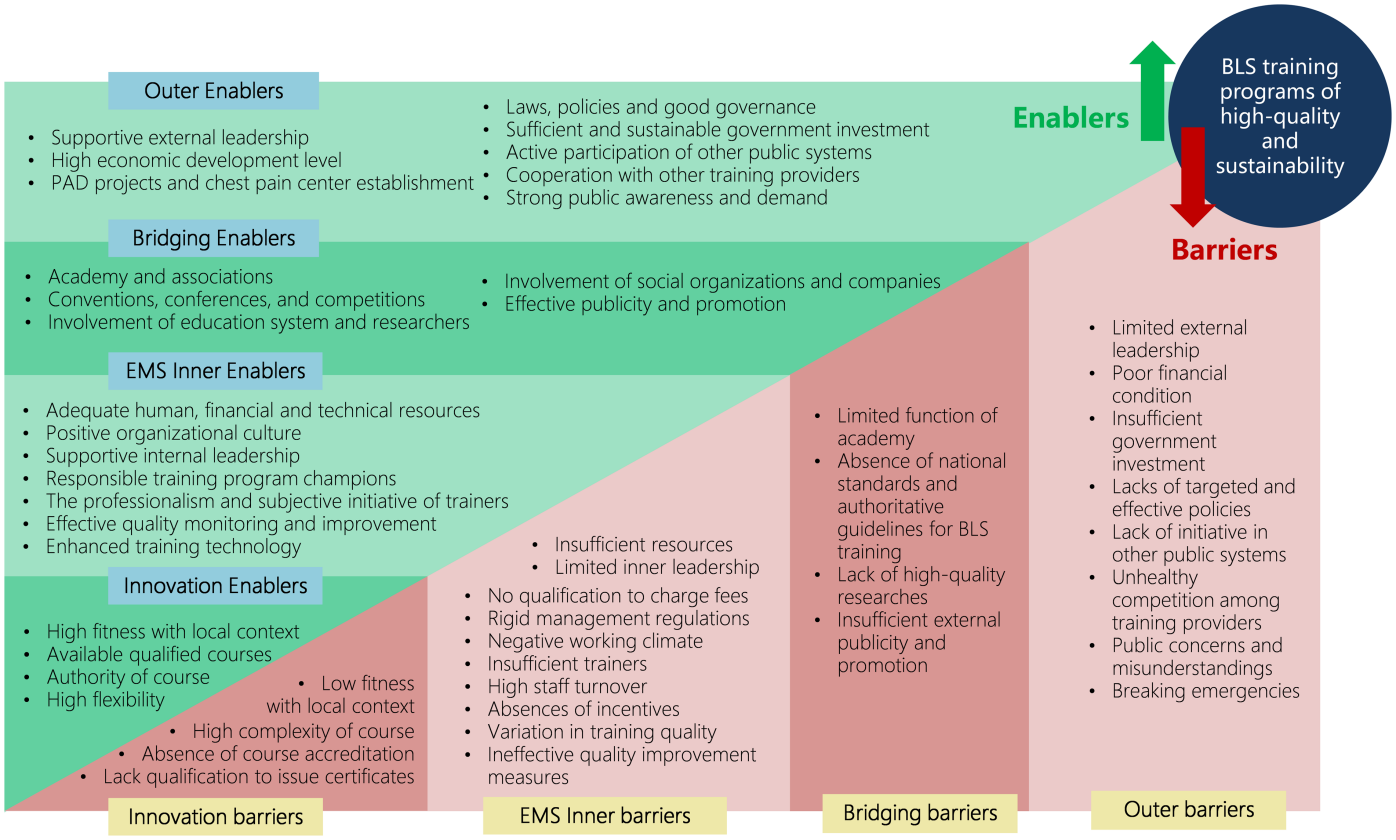
Applying the EPIS framework for factors influencing the implementation of BLS training programs for the public in EMS centers in China. EPIS, Exploration, Preparation, Implementation, Sustainment; BLS, basic life support; EMS, emergency medical service.

As shown in Figure 1, we identified 16 factors influencing the implementation of BLS training programs in 40 EMS centers based on the EPIS constructs. These factors encompass the outer context, inner context, innovation factors and bridging factors. Table 3 summarizes salient themes regarding each construct and indicates whether each influencing factor acted as a barrier or an enabler at each EPIS stage. Illustrative quotations were presented in Supplementary Table S2 to confirm the categories.

**Table 3:** Themes regarding implementation influences of BLS training programs in EMS centers.

Context	EPIS constructs	Implementation influences	Enabler (+) or Barrier (−) for each EPIS stage			
			E	P	I	S
Outer Context	Leadership	Government leadership	(+/−)	(+/−)	(+/−)	(+/−)
		Support of local health commission	(+/−)	(+/−)	(+/−)	(+/−)
		Social leadership	+	+	+	+
	Service environment/polices	Regional socioeconomic and demographic characteristics	(+/−)	(+/−)	(+/−)	(+/−)
		PAD projects and chest pain center establishment	+	+	+	+
		Major events, disasters, and pandemics	(+/−)	(+/−)	(+/−)	(+/−)
		Laws, policies, and governance	+	+	+	(+/−)
	Funding/Contracting	Government investment, budget, and funding	(+/−)	(+/−)	(+/−)	(+/−)
	Inter-organizational networks	Participation of other public systems and industries	+	+	+	(+/−)
		Co-opetition with other training providers	+	+	(+/−)	(+/−)
	Client advocacy/ characteristics	Public awareness and demand for BLS training	(+/−)	(+/−)	+	+
		Local culture and demographic characteristics	(+/−)	(+/−)	(+/−)	(+/−)
		The way of trainee involvement	(+/−)	(+/−)	(+/−)	(+/−)
Inner Context	Organizational characteristics	Personnel, equipment, supplies, technology resources	(+/−)	(+/−)	(+/−)	(+/−)
		EMS operation modes and supervision intensity	(+/−)	(+/−)	(+/−)	(+/−)
		Financial management requirements for EMS centers	(+/−)	(+/−)	(+/−)	(+/−)
		Organizational culture and working climate	(+/−)	(+/−)	(+/−)	(+/−)
	Leadership/champion	EMS center leadership	(+/−)	(+/−)	(+/−)	(+/−)
		Training department leaders and program champions	(+/−)	(+/−)	(+/−)	(+/−)
	Organizational staffing	BLS trainers’ number and staff turnover	(+/−)	(+/−)	(+/−)	(+/−)
		Personnel regime and incentive measures	(+/−)	(+/−)	(+/−)	(+/−)
	Individual characteristics	BLS trainers’ individual characteristics	(+/−)	(+/−)	(+/−)	(+/−)
		Experience in clinical, training, and certification	+	+	+	+
		Recognition and sense of responsibility	+	+	+	(+/−)
	Quality & Fidelity monitoring	Quality control and improvement measures	+	+	+	+
		Enhancement of smart technologies	+	+	+	+
		The authenticity of quality monitoring	+	+	+	(+/−)
Innovation factors	Innovation fit	Training content fit with local needs	(+/−)	(+/−)	(+/−)	(+/−)
		Training output fit with EMS system	(+/−)	(+/−)	(+/−)	(+/−)
		Training management fit with administrative system	(+/−)	(+/−)	(+/−)	(+/−)
	Innovation characteristics	The availability of qualified BLS courses	(+/−)	(+/−)	(+/−)	(+/−)
		The complexity and flexibility of BLS courses	(+/−)	(+/−)	(+/−)	(+/−)
		Cost-effectiveness considerations	(+/−)	(+/−)	(+/−)	(+/−)
	Innovation developer	The authority and reputation of EMS center	(+/−)	(+/−)	(+/−)	(+/−)
		The accreditation and qualification to issue certificates	(+/−)	(+/−)	(+/−)	(+/−)
Bridging factors	Community academic partnerships	Academy and associations of emergency medicine	(+/−)	(+/−)	(+/−)	(+/−)
		Cooperation with the education system and researchers	+	+	(+/−)	(+/−)
	Publicity and promotion	Publicity and promotion	(+/−)	(+/−)	(+/−)	(+/−)
	Purveyors/ intermediaries	Involvement of social organizations	+	+	+	+
		Support from manufacturers and technology companies	+	+	+	+

BLS, basic life support; EMS, emergency medical service; EPIS, Exploration, Preparation, Implementation, Sustainment; PAD, public access defibrillation.

### 3.1 Outer context

#### 3.1.1 Leadership

Leadership from outside of the EMS was particularly important throughout the EPIS stages, and a lack of leadership involvement or support was considered a major barrier. *Government leadership* could largely determine whether there were policies, resources, or funding targeted to BLS training programs. The *support of the municipal health commission*, to which the EMS center reports directly, also determined the funding, technology, human resources, and authorization of training programs. To date, the health commissions of most cities have not listed the amount or effect of BLS training as a performance indicator of EMS center, or the requirements lack specificity or binding effects.

In many cities, the *local People’s Congresses and People’s Political Consultative Conference*, which serve as legislatures and top political advisory bodies, have promoted BLS training for the lay public through actively proposing, promoting legislation, and supervising its implementation.

#### 3.1.2 Service environment/polices

*Regional socioeconomic and demographic characteristics,* such as economic development level, population size and age structure, were the outer context determinants in policy-making relevant to BLS training. Generally, cities with higher levels of economic development had better-established medical systems, higher government expenditures, and greater investments in health care and in EMS. Cities with large and aging populations placed greater emphasis on preventive measures including BLS training for the lay public, while sparsely populated cities faced challenges in implementing training programs.

The development of EMS, including the *initiation of PAD projects and the establishment of chest pain centers*, had greatly facilitated BLS training for the lay public. Cities’ experiences in hosting national and international events or conferences drove the optimization of EMS and the training of local volunteers. Disasters and sudden deaths of celebrities had raised public awareness of life-saving skills. The COVID-19 pandemic and its strict social distancing regulations necessitated had hindered the implementation of BLS training programs.

*Laws, policies, and governance* at the national and regional levels related to emergency healthcare, such as the Healthy China Initiative, the Good Samaritan law, provincial and municipal emergency medical regulations and livelihood projects, encouraged the promotion of training programs. However, most of the policies were noncoercive in nature. They only mandated that the healthcare system provide training, with no requirement for other systems or industries to participate in training, which was seen as a barrier at the sustainment stage.

#### 3.1.3 Funding/contracting of training

As a nonprofit public institution, the operation expenses of an EMS center rely largely on *government investment*. Insufficient funding was considered the greatest barrier for many EMS centers. In a few cities, governments have invested directly in purchasing training services from EMS centers. However, in most cities, EMS centers had to apply for special funding, and the approval of which might be uncertain. According to respondents, funding for BLS training was usually limited to short-term projects of 1–2 years, small in amount, and focused on “hardware” elements (e.g., EMS building, training site and equipment) rather than “software” aspects (e.g., course development and personnel expenses).

#### 3.1.4 Inter-organizational networks

*Public systems and industries* were encouraged to participate in BLS training programs offered by EMS centers, however, there were no mandatory training requirements for other public systems. *Cooperation between the EMS center and other training providers* would facilitate local BLS training coverage. Unfortunately, significant discrepancies in the content, forms, and fees of training offered by different providers had caused public confusion and market competition chaos, hindering the establishment of effective co-opetition.

#### 3.1.5 Client advocacy/characteristics

*Public awareness and demand* were crucial influencing factors for training implementation. As reflected by respondents, in the early years of training, low public awareness and reluctance of laypeople to receive training were the main barriers for them to promote BLS training programs. As public awareness and active participation in training had increased in recent years, public demand had been driving the development of training, and the training of many EMS center was in short supply.

The level of awareness and demand among the public was affected by *local cultural, regional, and individual characteristics*, including age, education level, and beliefs about life and death. In certain areas, particularly among the older adults, there might be concerns and misunderstandings about BLS training due to superstitions about discussing death, worries about potential harm caused by CPR, doubts about the effectiveness of BLS skills in saving their own lives, or hesitance to kneel during CPR.

The *way of trainee involvement* also affected the training effect. Compared with those who actively paid for their courses, trainees who engage in free training activities or participated in task-oriented training organized by their company tended to have less initiative and lower training effectiveness.

### 3.2 Inner context

#### 3.2.1 Organizational characteristics

*Organizational characteristics* of an EMS center determined the amount of personnel, equipment, supplies, technology, and funding required for training programs. These characteristics included the existence of training departments, dedicated trainers, venues, and certifications as centers for standardized courses, and could vary greatly among different *EMS operation modes*. Generally, the independent and prehospital mode EMS centers were large in scale and had adequate venues, training department and trainers. On the other hand, the dispatching and dependent mode EMS centers usually had no trainer or venue to provide training to public and relied heavily on resources from network hospitals. In such cases, the *intensity of supervision and guidance* of EMS center on network hospitals would affect the implementation. For instance, a municipal level EMS center might not be able to enforce strict requirements on provincial or ministerial hospitals in the same city due to administrative issues.

The financial strain had limited the implementation of training. The *nonprofit nature of EMS centers* prevented them from charging fees for training services. While some EMS centers were able to charge reasonable fees under government supervision, these funds could not be allocated directly to training due to the *separation of revenue and expenditure budgets*.

*The organizational culture and working climate* would influence the readiness of EMS centers to implement training programs. A positive culture, characterized by the will to grasp the nettle, the courage to change, an open-minded and enterprising attitude, enabled implementation. Conversely, a hidebound mind, rigid thinking, and benefit-oriented climate acted as barriers.

#### 3.2.2 Leadership and champion

The *leadership of EMS centers* determined the approach, form, and scope of training programs. Although the leaders of all EMS centers were supportive of training for the lay public, there were differences in how they enforced it. A change in leadership would also challenge the continuation and sustainability of training programs and policies.

The professionalism, working philosophy and capability of the *training department leaders* ensured both the quality and quantity of training. What truly distinguished *training program champions*, who were engaging, influential and devoted for the promotion of the program, apart from others was their sense of responsibility and mission.

#### 3.2.3 Organizational characteristics organizational staffing

The *number of BLS trainers* in the EMS center was a strong determinant of the efficiency and difficulty of training implementation. There was a general shortage of trainers in EMS centers, and high *staff turnover and attrition* also posed challenges.

Compared with doctors from other specialties, EMS personnel generally received lower salaries and less social recognition. Due to the *personnel regime* of public institutions, BLS trainers received fixed compensation, with no performance-based pay for training. All respondents acknowledged the lack of *incentive measures* for trainers and expressed concern that this could lead to a decline in quality and long-term unsustainability.

#### 3.2.4 Individual characteristics

The quality and effectiveness of training courses conducted by different trainers varied and were influenced by the *trainer’s individual characteristics*, such as age, educational level, personality, and language/local dialect skills. The trainers’ teaching skills could be enhanced through their *experience in clinical treatment, training and certification, practical course exercises, and EMS skills competitions*. Their *feelings, beliefs, recognition, and sense of responsibility* toward the training program determined their level of subjective initiative.

#### 3.2.5 Quality and fidelity monitoring

In response to the wide disparities in teaching quality among individual trainers, EMS centers generally adopted a variety of *quality control and improvement measures*. These measures could be summarized as: (1) strict course design and review, (2) unification and standardization of course materials, (3) whole-process management of trainees, and (4) training, certification, supervision, and assessment of trainers. Some EMS centers had issued management regulations or standards and adopted third-party assessments to ensure the fidelity of quality control approaches. The *development of smart technologies*, such as smartphones, surveillance cameras, and feedback devices, had facilitated quality monitoring. However, there are still instances of noncompliance with regulations. Some *quality monitoring indicators* that had remained unchanged for years could no longer truly reflect the quality, and staff turnover hindered quality maintenance.

### 3.3 Innovation factors

#### 3.3.1 Quality and fidelity monitoring innovation fit

The *fit between the training and local context* was crucial, as it was reflected in three aspects: the content fit with the local citizens, the training output fit with the EMS system, and the training management fit with the local administrative system. The content should be localized, easy for lay people to understand and memorize, address common concerns, and adaptable to the needs and feedback of trainees. To fit in within the EMS system, local EMS protocols should be introduced, and certified trainees should be incorporated into a volunteer system that could be dispatched by the EMS center. Unfortunately, there had been insufficient integration between the training system, volunteer system and dispatching system.

#### 3.3.2 Innovation characteristics

*The availability of well-designed qualified BLS courses* was fundamental to the implementation of training programs. There were 3 main types of courses provided by EMS centers: (1) American Heart Association (AHA)/European Resuscitation Council (ERC)/ other standardized productionized courses, (2) courses developed by the national emergency medical association or local health committee, (3) courses developed by the EMS centers themselves. Due to financial constraints, most EMS centers opted to create their own courses. However, some found this difficult without industry authoritative guidance or national standards for BLS training. Furthermore, the wide variation in BLS course content, format, length, quality among cities had led to public confusion and questioning of authority. Respondents generally advocated for the establishment of national standards for BLS courses and accreditation.

To ensure the high quality of BLS training, EMS centers attached great importance to interaction and practical exercises in their courses, which required sufficient trainers, manikins, and other training devices and increased the *complexity* of course implementation. Some EMS centers adopted innovative strategies, such as micro-training stations, remote or online training methods, to improve the *flexibility*. Additionally, the implementation of whole-process management and systematic retraining imposed higher requirements, and had been realized only in a few EMS centers.

*Cost-effectiveness considerations* influenced the implementation of BLS training. According to some respondents, there were instances when their superiors were unsatisfied with the training efficiency of small-class teaching. They had to resort to a more ‘efficient’ approach by reducing the ratio of trainers and manikins to trainees, or by shortening the practice time. This was clearly contrary to the requirements of high-quality training. Regarding fees, the respondents believed that different charging fees for BLS training for the public would create chaos in the market. They argued that free courses would lead the public to believe that BLS training had no value. None of these would contribute to the sustainable development of the industry.

#### 3.3.3 Innovation developers

The *authority of the training provider* affected the public recognition and reputation of the training. Except for a few EMS centers that had been authorized by local health commission to provide training, most self-developed courses lacked accreditation. Although bystanders do not need to hold a certificate to save lives, many institutions and citizens still desire a BLS certificate. Unfortunately, many EMS centers had to reject these potential trainees because they were not *qualified to issue certificates*, which was seen as a major barrier to the implementation and sustainability of training programs.

### 3.4 Bridging factors

#### 3.4.1 Community academic partnerships

The *national academy and associations of emergency medicine* had continuously promoted the implementation of BLS training programs across the country. They had led the formation of industry consensuses, created BLS courses, and provided national platforms for experience sharing and learning. Unfortunately, they had not yet put forward authoritative guidelines for BLS education that EMS centers could refer to.

*Cooperation with the education system and researchers* was an enabler of training programs. On the one hand, local education departments and schools at all levels had paid increasing attention to BLS training for students, expanding the coverage of training. On the other hand, the initiation of researchers had steered local training programs to a more scientific and innovative direction. However, there was still a shortage of high-level talent and high-quality research in this field.

#### 3.4.2 Publicity and promotion

EMS centers were generally faced with obstacles caused by insufficient *publicity and promotion*. Their publicity and promotion activities encompassed both daily and event-based types. For daily publicity, EMS centers primarily utilized platforms such as TV, newspapers, social media, and public transportation to disseminate key concepts to the public, including the Good Samaritan Law, how to perform CPR and use AEDs. The promotional events were mainly held on memorial days such as the 120 First Aid Day, the CPR week, the World Restart a Heart Day, etc. to raise public awareness about BLS. Most of the publicity was done by training providers themselves, with limited presence of external long-term channels. The focus of content was on raising awareness and knowledge about BLS skills, rather than informing the public about where they could receive training. Traditional media was the primary means of publicity, but it failed to reach all age groups effectively. Moreover, the publicity efforts were not always standardized and were often contradictory, which hindered expansion. Reasons for this inadequate publicity included high cost, insufficient investment, lack of relevant staff, and limited leadership attention.

#### 3.4.3 Purveyors/intermediaries

The *involvement of social organizations*, such as volunteer associations and industry groups, was conducive to the training implementation, and promoted the publicity and impact of BLS training. Some *manufacturers and distributors of training equipment, AED companies, Internet and smart technology companies* had supported the development of training through investing in equipment, providing technical supplements, and constructing technical platforms.

## 4 Discussion

### 4.1 Main findings

**Figure 2:**
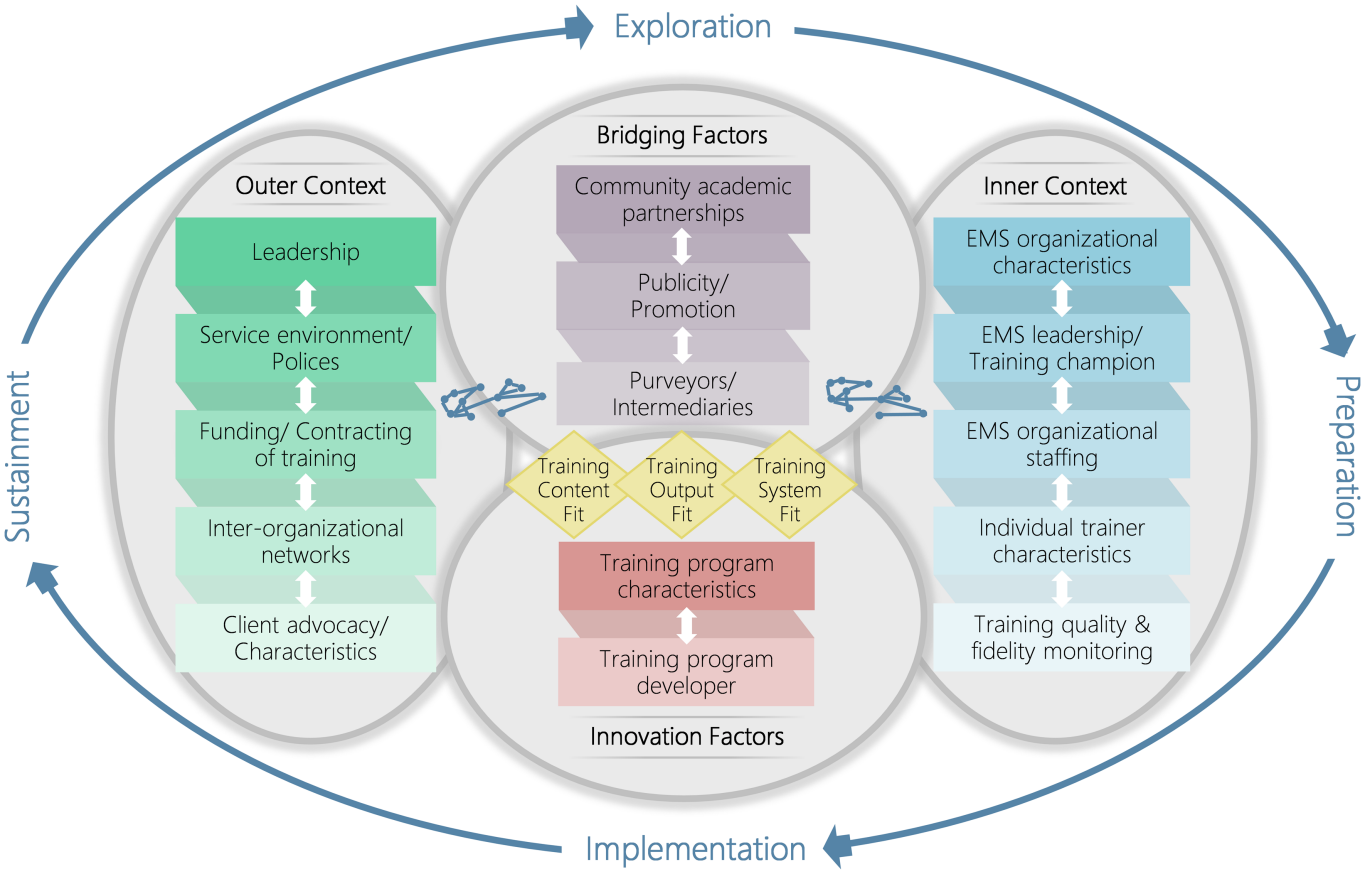
Barriers and enablers to the implementation of BLS training programs for the lay public in EMS centers in China. BLS, basic life support; EMS, emergency medical service; PAD, public access defibrillation. Reproduced from Moullin et al. (26), licensed under Creative Commons Attribution 4.0 International License.

This is the first study to provide in-depth information on the factors influencing the implementation of BLS training for the lay public at a national level in China. Applying the EPIS framework, we described and identified barriers and enablers that need to be addressed to ensure the implementation of effective and sustainable BLS training programs (Figure 2). These factors included the outer, inner, bridging and innovation contexts, and had different impacts at different stages of the EPIS.

As a social welfare project, the government is the investor ‘pushing’ the BLS training program through policy and funding, setting the lower limit of training amount. The public, on the other hand, is the client ‘pulling’ the program with their demand, and determining the upper limit. The EMS center acts as the government’s way of serving the public, and its inner factors determine the efficiency of training. While the public demand for training has increased rapidly in recent years, the government investment has not increased in parallel. This has resulted in EMS centers being unable to meet the public demand with current efficiency. Government investment is mainly in equipment rather than human resources, and lacks certainty and sustainability. Despite an overall increase in public demand, there are still large regional disparities, and ongoing public concerns about BLS training. These issues demand attention and need to be addressed.

The EMS inner factors are salient in the implementation process, and can be categorized into input, personnel, and system dimensions. In terms of input, the resources of the EMS centers are sourced from government investment, which makes it difficult to be affected by inner factors in short term. For personnel dimension, the nonprofit nature of public institutions limits the incentives for trainers, resulting in a decline in their subjective initiative and high turnover rate. In fact, this problem is not restricted to training departments alone, as previous literatures have reported a general challenge in staffing of EMS system (20, 21, 31). While the EMS system is progressing in a more scientific and efficient direction, it remains challenging for EMS centers to improve and maintain their internal capacity due to a lack of input and personnel.

Factors related to the innovation itself, such as the complexity of BLS course and its adaptation to the local context, are highlighted. The professionalism and work experiences ensure the quality and fit of EMS training courses, however, the lack of authoritative guidelines and qualifications for courses severely hinders the implementation and promotion of EMS training. Respondents strongly emphasized the need for national BLS training guidelines. Some suggest the establishment of a unified national course system, while others believe that unified certification and accreditation of courses would be more realistic. Although the considerations in the approaches differ, all agree that this is an urgent issue that can be addressed only through top-level design.

Certain factors acted as either barriers or enablers at different stages of BLS training implementation. Public awareness and demand served as a barrier in the early years but is now an enabler. For example, the Beijing EMS center reported a marked increase in public requests for training after the 2008 Olympics, and the turning point for Shanghai and Shenzhen was the launch of PAD programs (33, 34). A similar trend has been seen in other regions where mandated policies have transformed into subjective initiatives, such as in Denmark, Norway, and Australia (35–37). Conversely, some factors, such as laws and policies, participation of other public systems, the authenticity of quality control measures, and cooperation with the education system and researchers, were initially enablers but are now hindering the sustainability. The reasons might be, as we believe, the lack of long-term mechanisms, specificity, and comprehensive policies. Implementation of interventions is meaningless without successful long-term use (29), and our findings imply potential areas for improving sustainability.

It is a systematic program of the whole society to provide BLS education to the lay public. To achieve the public health goal that everyone receives BLS training to raise bystander CPR rates and thereby increase survival after OHCA requires a joint effort from all stakeholders. It is equally important to address the translation of training to local community response (local implementation) as well as medical science and educational efficiency (8). This is a process from quantitative change to qualitative change. At present, the focus of BLS training is still on expanding the quantity rather than effectively involving trained laypeople as active community first responders. Only a few cities have developed volunteer systems and empowered them through dispatch.

### 4.2 Implications for practice and policy

To facilitate the implementation of BLS training for the lay public, improvements can be made at the national level, city level, and EMS institutional level.

Two key issues must be addressed at national level. First, it is paramount to promote legislation and policies regarding BLS education. The legislation should be more targeted, with specific requirements for both the providers and demanders. Supporting policies should be in place to ensure adequate resources, publicity, and enforcement of monitoring measures. Second, there is an urgent need to establish unified BLS training standards and guidelines, as well as to conduct accreditation of BLS curricula. This process should involve all stakeholders in order to reach a top-level consensus and ensure its feasibility.

City governments need to carefully consider and prioritize larger, more sustained, and better-balanced investments in EMS centers. They should take the lead in organizing, coordinating, supervising, and publicizing BLS training programs. It is also crucial for government and health commissions to authorize local BLS curricula and provide quality supervision.

At the institutional level, EMS centers should continuously strive to enhance and maintain the professionalism, appropriateness, and quality of their training courses. This study clearly demonstrated the need for incentives for BLS trainers, and for involving trained laypeople in active community responses. The collaboration between EMS center with other training providers, other public systems and local communities should be strengthened. Additionally, emphasis should be placed on effective data analysis and scientific researches, as they are the foundational work toward a culture of excellence (24).

In comparison to some successful projects in other countries, the outstanding feature of the BLS training projects of EMS centers in China is that they are led and funded by the government. While basic coverage and certain equity have been achieved, the efficiency of training has been constrained by limited funding’s and human resources. In contrast, training programs in some countries, such as the Nation of Lifesavers, HEROS, and DARE, are primarily funded by foundations with more flexible and sufficient financial support. Their highly standardized training course products, which are always 1–2 h in length, could be utilized in conjunction with the train-the-trainer model to train school students and public employees. These measures to improve the cost-efficiency of BLS training could be used as a reference.

### 4.3 Limitations

This study has several limitations. First, it examined the implementation of BLS training only from the urban EMS perspective, thus, the findings may not represent the situation of other training providers in China, and may not apply to other settings. Second, our interviews included only key informants from EMS centers and did not include viewpoints from other stakeholders, such as the government, health commission, or public. This may lead to our overestimation or underestimation of some influencing factors. Third, the participating EMS centers are primarily located in municipalities and provincial capitals with more resources and better development conditions than those in smaller cities and rural areas, which may limit the external validity of the results. Certain factors may have different effects on EMS centers of different regions, and need to be further explored. Furthermore, this was a descriptive qualitative research, which limits our ability to confirm whether the identified factors are indeed associated with training impact. Nevertheless, this national-representative study involved key informants from 40 EMS centers across China and focused on the implementation of training programs at institutional and individual levels. Our findings highlighted both successful experiences and reasons for unsuccessful implementation, providing valuable reference for training providers and policymakers to facilitate the implementation of BLS training for the lay public.

## 5 Conclusion

Based on the EPIS framework, this study recognized outer, inner, innovation and bridging factors influencing implementation of BLS training programs in EMS centers in Chinese cities, and identified barriers and enablers throughout the implementation process. Our findings highlight the diversity of stakeholders, the complexity of implementation, and the need for localization and co-construction. To facilitate the implementation of BLS training for lay public, efforts should be made at the national level, city level, and EMS institutional level to boost priority and awareness, promote legislation and policies, raise sustainable resources, and enhance the technology of BLS courses.

## Data availability statement

The data that support the findings of this study are available from the corresponding author upon reasonable request.

## Ethics statement

The studies involving humans were approved by the Joint Research Ethics Board of the Shanghai Jiao Tong University Schools of Public Health and Nursing (SJUPN-HY-202304-5-KS1). The studies were conducted in accordance with the local legislation and institutional requirements. Written informed consent to participate in this study was not required from the participants in accordance with the national legislation and the institutional requirements. Verbal informed consent was obtained from all respondents before the interviews.

## Author contributions

XD: Conceptualization, Data curation, Formal analysis, Investigation, Visualization, Writing – original draft, Writing – review & editing. LZ: Conceptualization, Funding acquisition, Investigation, Resources, Supervision, Writing – review & editing. ZW: Writing – review & editing. Z-jZ: Conceptualization, Resources, Supervision, Validation, Writing – review & editing.

## References

[1] Kiguchi T Okubo M Nishiyama C Maconochie I Ong MEH Kern KB. Out-of-hospital cardiac arrest across the world: first report from the international liaison committee on resuscitation (ILCOR). Resuscitation. (2020) 152:39–49. doi: 10.1016/j.resuscitation.2020.02.044, PMID: 32272235

[2] Wyckoff MH Singletary EM Soar J Olasveengen TM Greif R Liley HG. 2021 international consensus on cardiopulmonary resuscitation and emergency cardiovascular care science with treatment recommendations: summary from the basic life support; advanced life support; neonatal life support; education, implementation, and teams; first aid task forces; and the COVID-19 working group. Circulation. (2022) 145:e645–721. doi: 10.1161/CIR.0000000000001017, PMID: 34813356

[3] Ong MEH Perkins GD Cariou A. Out-of-hospital cardiac arrest: prehospital management. Lancet (London, England). (2018) 391:980–8. doi: 10.1016/S0140-6736(18)30316-7 29536862

[4] Jensen TW Ersbøll AK Folke F Wolthers SA Andersen MP Blomberg SN. Training in basic life support and bystander-performed cardiopulmonary resuscitation and survival in out-of-hospital cardiac arrests in Denmark, 2005 to 2019. JAMA Netw Open. (2023) 6:e233338. doi: 10.1001/jamanetworkopen.2023.3338, PMID: 36929397 PMC10020888

[5] Jensen TW Ersbøll AK Folke F Andersen MP Blomberg SN Holgersen MG. Geographical association between basic life support courses and bystander cardiopulmonary resuscitation and Survival from OHCA in Denmark. Open access emerg med: OAEM. (2023) 15:241–52. doi: 10.2147/OAEM.S405397, PMID: 37342237 PMC10278866

[6] Malta Hansen C Kragholm K Pearson DA Tyson C Monk L Myers B. Association of Bystander and First-Responder Intervention with Survival after out-of-Hospital Cardiac Arrest in North Carolina, 2010-2013. JAMA. (2015) 314:255–64. doi: 10.1001/jama.2015.7938, PMID: 26197186

[7] Son JW Ryoo HW Moon S Kim JY Ahn JY Park JB. Association between public cardiopulmonary resuscitation education and the willingness to perform bystander cardiopulmonary resuscitation: a metropolitan citywide survey. Clinical experimental emerg med. (2017) 4:80–7. doi: 10.15441/ceem.16.160, PMID: 28717777 PMC5511954

[8] Søreide E Morrison L Hillman K Monsieurs K Sunde K Zideman D. The formula for survival in resuscitation. Resuscitation. (2013) 84:1487–93. doi: 10.1016/j.resuscitation.2013.07.020 23917078

[9] Cheng A Nadkarni VM Mancini MB Hunt EA Sinz EH Merchant RM. Resuscitation education science: educational strategies to improve outcomes from cardiac arrest: a scientific statement from the American Heart Association. Circulation. (2018) 138:e82–e122. doi: 10.1161/CIR.0000000000000583, PMID: 29930020

[10] Schnaubelt S Greif R Monsieurs K. The chainmail of survival: a modern concept of an adaptive approach towards cardiopulmonary resuscitation. Resuscitation. (2023) 184:109707. doi: 10.1016/j.resuscitation.2023.109707, PMID: 36709826

[11] Dong X Kong SYJ Xu H Ho AFW Blewer AL Birkenes TS. "needed but lacked": exploring demand-and supply-side determinants of access to cardiopulmonary resuscitation training for the lay public in China. Front Public Health. (2023) 11:1164744. doi: 10.3389/fpubh.2023.1164744, PMID: 37124786 PMC10130457

[12] Tiwari L Lockey A Böttiger BW Rott N Hoover AV Chakra Rao S. More than 302 million people reached and over 2,200,000 trained in cardiopulmonary resuscitation worldwide: the 2021 ILCOR world restart a heart initiative. Resuscitation plus. (2023) 14:100375. doi: 10.1016/j.resplu.2023.100375, PMID: 37007185 PMC10060744

[13] Schnaubelt S Monsieurs KG Semeraro F Schlieber J Cheng A Bigham BL. Clinical outcomes from out-of-hospital cardiac arrest in low-resource settings - a scoping review. Resuscitation. (2020) 156:137–45. doi: 10.1016/j.resuscitation.2020.08.126 32920113

[14] Perkins GD Lockey AS de Belder MA Moore F Weissberg P Gray H. National initiatives to improve outcomes from out-of-hospital cardiac arrest in England. Emerg Med J. (2016) 33:448–51. doi: 10.1136/emermed-2015-204847, PMID: 26400865 PMC4941191

[15] Naccarella L Saxton D Lugg E Marley J. It takes a community to save a life in cardiac arrest: heart safe community pilots, Australia. Health Promotion J Australia: Official J Australian Association of Health Promotion Professionals. (2022) 33:99–105. doi: 10.1002/hpja.482 33743556

[16] Park GJ Kong SYJ Song KJ Shin SD Kim TH Ro YS. The effectiveness of a new dispatcher-assisted basic life support training program on quality in cardiopulmonary resuscitation performance during training and willingness to perform bystander cardiopulmonary resuscitation: a cluster randomized controlled study. Simulation in healthcare: J Society for Simulation in Healthcare. (2020) 15:318–25. doi: 10.1097/SIH.0000000000000435, PMID: 32604135

[17] Blewer AL Ho AFW Shahidah N White AE Pek PP Ng YY. Impact of bystander-focused public health interventions on cardiopulmonary resuscitation and survival: a cohort study. Lancet Public Health. (2020) 5:e428–36. doi: 10.1016/S2468-2667(20)30140-7, PMID: 32768435

[18] Zheng J Lv C Zheng W Zhang G Tan H Ma Y. Incidence, process of care, and outcomes of out-of-hospital cardiac arrest in China: a prospective study of the BASIC-OHCA registry. Lancet Public Health. (2023) 8:e923–32. doi: 10.1016/S2468-2667(23)00173-1, PMID: 37722403

[19] Xu F Zhang Y Chen Y. Cardiopulmonary resuscitation training in China: current situation and future development. JAMA Cardiol. (2017) 2:469–70. doi: 10.1001/jamacardio.2017.0035, PMID: 28297007

[20] Hung KK Cheung CS Rainer TH Graham CA. EMS systems in China. Resuscitation. (2009) 80:732–5. doi: 10.1016/j.resuscitation.2009.04.016, PMID: 19443099

[21] Pei YV Xiao F. Emergency medicine in China: present and future. World J Emerg Med. (2011) 2:245–52. doi: 10.5847/wjem.j.1920-8642.2011.04.001, PMID: 25215018 PMC4129727

[22] Kragholm K Wissenberg M Mortensen RN Hansen SM Malta Hansen C Thorsteinsson K. Bystander efforts and 1-year outcomes in out-of-hospital cardiac arrest. N Engl J Med. (2017) 376:1737–47. doi: 10.1056/NEJMoa1601891, PMID: 28467879

[23] Scapigliati A Zace D Matsuyama T Pisapia L Saviani M Semeraro F. Community initiatives to promote basic life support implementation-a scoping review. J Clin Med. (2021) 10:5719. doi: 10.3390/jcm10245719, PMID: 34945015 PMC8703423

[24] Global Resuscitation Alliance. Improving Survival from Out-of-Hospital Cardiac Arrest Acting on the Call. (2018). Available online at: https://www.globalresuscitationalliance.org/wp-content/pdf/acting_on_the_call.pdf.

[25] Resuscitation academy. Toolkits: THE RESUSCITATION ACADEMY; (2021). Available from: https://www.resuscitationacademy.org/toolkits.

[26] Moullin JC Sabater-Hernández D Fernandez-Llimos F Benrimoj SI. A systematic review of implementation frameworks of innovations in healthcare and resulting generic implementation framework. Health Res Policy Syst. (2015) 13:16. doi: 10.1186/s12961-015-0005-z, PMID: 25885055 PMC4364490

[27] Aarons GA Hurlburt M Horwitz SM. Advancing a conceptual model of evidence-based practice implementation in public service sectors. Admin Pol Ment Health. (2011) 38:4–23. doi: 10.1007/s10488-010-0327-7, PMID: 21197565 PMC3025110

[28] Moullin JC Dickson KS Stadnick NA Rabin B Aarons GA. Systematic review of the exploration, preparation, implementation, sustainment (EPIS) framework. Implementation science: IS. (2019) 14:1. doi: 10.1186/s13012-018-0842-6, PMID: 30611302 PMC6321673

[29] Chambers DA Glasgow RE Stange KC. The dynamic sustainability framework: addressing the paradox of sustainment amid ongoing change. Implement Sci. (2013) 8:117. doi: 10.1186/1748-5908-8-117, PMID: 24088228 PMC3852739

[30] O’Brien BC Harris IB Beckman TJ Reed DA Cook DA. Standards for reporting qualitative research: a synthesis of recommendations. Acad Med. (2014) 89:1245–51. doi: 10.1097/ACM.0000000000000388 24979285

[31] Shi X Bao J Zhang H Wang H Wang Y Li L. Emergency medicine in China: a review of the history of progress and current and future challenges after 40 years of reform. Am J Emerg Med. (2020) 38:662–9. doi: 10.1016/j.ajem.2019.11.008, PMID: 31902696

[32] Dong X Ding F Zhou S Ma J Li N Maimaitiming M. Optimizing an emergency medical dispatch system to improve prehospital diagnosis and treatment of acute coronary syndrome: Nationwide retrospective study in China. J Med Internet Res. (2022) 24:e36929. doi: 10.2196/36929 36416876 PMC9730207

[33] Global Resuscitation Alliance. Public access defibrillation program in Shenzhen, China. (2019). Available online at: https://www.globalresuscitationalliance.org/wp-content/uploads/2019/12/China_PAD.pdf.

[34] Zhang L Li B Zhao X Zhang Y Deng Y Zhao A. Public access of automated external defibrillators in a metropolitan city of China. Resuscitation. (2019) 140:120–6. doi: 10.1016/j.resuscitation.2019.05.015, PMID: 31129230

[35] Juul Grabmayr A Andelius L Bo Christensen N Folke F Bundgaard Ringgren K Torp-Pedersen C. Contemporary levels of cardiopulmonary resuscitation training in Denmark. Resuscitation plus. (2022) 11:100268. doi: 10.1016/j.resplu.2022.100268, PMID: 35812720 PMC9256815

[36] Bakke HK Steinvik T Angell J Wisborg T. A nationwide survey of first aid training and encounters in Norway. BMC Emerg Med. (2017) 17:6. doi: 10.1186/s12873-017-0116-7, PMID: 28228110 PMC5322636

[37] Cartledge S Saxton D Finn J Bray JE. Australia's awareness of cardiac arrest and rates of CPR training: results from the Heart Foundation's HeartWatch survey. BMJ Open. (2020) 10:e033722. doi: 10.1136/bmjopen-2019-033722 PMC6955479 31911523

