# Supplementary material for: Implementation of basic life support education for the lay public in China: barriers, enablers, and possible solutions
Source: Front Public Health. 2024 Jun 27;12:1390819. doi: 10.3389/fpubh.2024.1390819 (PMC11236690; doi:10.3389/fpubh.2024.1390819)
Supplement: Supplementary file 1 [file Data_Sheet_1.pdf]

Supplementary Material

1 Supplementary Table 1

Table S1. Main operation modes of EMS centers in Chinese cities

Mode	Function		
	**Dispatching**	**Pre-hospital transport**	**In-hospital treatment**
Independent mode	EMS	EMS	EMS
Prehospital mode	EMS	EMS	Network hospitals
Dispatching mode	EMS	Network hospitals	Network hospitals
Dependent mode	The hospital that EMS center affiliated to	Network hospitals	Network hospitals

EMS- emergency medical service.

2 Supplementary Table 2

Table S2. Themes and quotes regarding implementation influence of BLS training programs in EMS centers

Context	EPIS constructs	Implementation influences	Verbatim quotes
**Outer Context**	Leadership	Government leadership	*"The key lies at the government. The government can support from several aspects: money, policy, and providing public platform. The awareness of local government determines whether the training program goes well or not."* (D10, male, 59yrs, EMS center director, east region, prefecture-level city)
		Support of local health commission	*"The most important enabler is the support from the health system. If there wasn't a first aid regulation, if there wasn't support from our health commission, the finance department wouldn't know about BLS training for the lay public, and there wouldn't be funding for us to do it."* (D6, male, 39yrs, EMS center deputy director, central region, provincial capital city)<br><br>*"In fact, I think there is not enough support from all levels. The city government does not really care about training, and the health commission does not have a clear goal. Due to our limited manpower, our center will not give you a clear request either."* (L20, male, 50yrs, training department leader, west region, provincial capital city)
		Social leadership	*"The promotion of the government is also related to the constant appeals of democratic parties and the CPPCC. For years, they have been proposing first aid regulations, public BLS training and AEDs."* (D3, male, 59yrs, EMS center director, east region, prefecture-level city)
	Service environment/ polices	Service environment	*"The most important influencing factor should be economic and social development. Only after the economic development will people have the time and energy to take care of their own and others' health, and will be willing to learn BLS skills. And the government will have the financial resources to support us to do publicity and training. Third, the government will put AED in public places, then enterprises will buy AEDs."* (D11, male, 43yrs, EMS center deputy director, east region, provincial capital city)<br><br>*"I believe that the level of EMS development equals the level of economic development in a city. We can feel that the southern region gets more investment. For us, the economy is relatively backward*

			across the country, the overall economy is not good, and the investment in EMS is relatively low." (L10, male, 48yrs, training department leader, central region, provincial capital city)
		PAD projects and chest pain center establishment	*"The PAD program has definitely promoted our training. People can see the AEDs in the city, they get curious. And the AED manufactures have a duty to train people around AED, which also promotes my work."* (L24, male, 55yrs, training department leader, east region, provincial capital city)<br><br>*"In fact, the BLS training for the public, the construction of chest pain centers, stroke centers, and trauma centers are mutually reinforcing."* (D7, female, 51yrs, EMS center deputy director, west region, provincial capital city)
		Major events, disasters, and pandemics	*"I remember that our training department was set up after the Beijing Olympics, which was a good opportunity for public BLS training."* (L13, male, 48yrs, training department leader, east region, provincial capital city)<br><br>*"We started the PAD and mass training in our city after holding international marathons."* (L2, female, 46yrs, training department leader, central region, provincial capital city)<br><br>*"The COVID-19 pandemic broke out shortly after the establishment of our training center, which to some extent affected the development of training."* (D6, male, 39yrs, EMS center deputy director, central region, provincial capital city)
		Laws, policies, and governance	*"Since the Healthy China Initiatives launched in 2019, the public training has gradually been valued by the government and our leaders."* (L4, male, 39yrs, training department leader, east region, provincial capital city)<br><br>*"Many cities have local first aid regulations, which include public BLS training. However, I think it is regrettable that, although there are some policy guidelines, there is not a rigid requirement."* (T4, male, 45yrs, senior trainer, east region, provincial capital city)
	Funding/Contracting	Government investment, budget, and funding	*"The main barrier is limited budget. If we can receive more investment from the government, we should be able to work more smoothly."* (L14, female, 53yrs, training department leader, west region, centrally-administered municipality)<br><br>*"In some cities with better performance, they are funded directly by the government. For us, the government support is only through policy and there is no direct financial investment."* (L18, male, 44yrs, training department leader, east region, centrally-administered municipality)

	Inter-organizational networks	Participation of other public systems and industries	*"Generally, other public systems, such as fire department, electricity, power grids, come to us for training. But this is not yet a regulation or a system."* (D5, male, 51yrs, EMS center deputy director, west region, prefecture-level city)
		Co-opetition between EMS center and other training providers	"*At present, the main providers of training are the Red Society, some rescue organizations, and us. I can say that our training is the most professional one, and the actual quality of training in some other institutions may be poor.*" (L5, male, 44yrs, training department leader, east region, centrally-administered municipality)<br><br>"*Unfortunately, the training providers are not working together. For us, we hope to integrate all trained laypeople into our volunteer system through volunteers, but it is difficult.*" (L24, male, 55yrs, training department leader, east region, provincial capital city)
	Client advocacy/ characteristics	Public awareness and demand for BLS training	*"I think the public's acceptance of BLS training is growing. In the early days, people were not acceptable of the training, thinking, "What is the use of me learning this?". In recent years, the proportion of people who take the initiative to learn and find us is getting higher and higher, and now we may sometimes be in a state of being too busy."* (L18, male, 44yrs, training department leader, east region, centrally-administered municipality)<br><br>*"There is a big market, I think people have a lot of demand, greater than what we have done now. The difficulty is that, mass training is not a department's thing. We need to get the trainee to take the initiative, and make them believe that it is meaningful."* (L15, male, 40yrs, training department leader, east region, centrally-administered municipality)
		Local culture, regional and individual demographic characteristics	*"The education level of trainees may affect the learning ability, and many people are unwilling to do practical CPR exercises because they refuse to kneel."* (D7, female, 51yrs, EMS center deputy director, west region, provincial capital city)<br><br>*"Some trainees are learning with a sad state of mind, they even cry while they practicing. The past accident happened to their families or friends drive them to learn BLS, and moved us a lot."* (L11, male, 46yrs, training department leader, central region, provincial capital city)
		The way of trainee involvement	"*There are many types of students. If it is a public course that we charge, people sign up for it themselves, they may be the most motivated. There are also some voluntary activities that we do, the citizen does not spend money, but they are willing to spend time to learn. However, if the course is*

			arranged by leaders of some companies, the participants tend to pay less attention to it." (D11, male, 43yrs, EMS center deputy director, east region, provincial capital city)
**Inner Context**	Organizational characteristics	Personnel, equipment, supplies, technology resources	*"We are attached to the downtown hospital, so our emergency center has no training venue, which is the biggest problem for me. Most of my training involves traveling to various places. Or just borrow a conference room at the hospital, but it's not suitable for training."* (L10, male, 48yrs, training department leader, central region, provincial capital city)
		EMS operation modes and supervision intensity	*"Some EMS centers have financial support and personnel, but some centers only have dispatchers and lack of doctors and nurses to provide training."* (D11, male, 43yrs, EMS center deputy director, east region, provincial capital city)<br><br>*"Our center is direct under the municipal government, but we are in the provincial capital city, that is, many network hospitals are leveled higher than us. It'll be hard for us to manage them."* (L12, female, 38yrs, training department leader, west region, provincial capital city)
		Financial management requirements for EMS centers	"*Most EMS centers are fully funded public institutions, which leads to two problems. They cannot charge training fees, and they cannot pay trainers labor fees, which affects the enthusiasm of personnel a lot.*" (D11, male, 43yrs, EMS center deputy director, east region, provincial capital city)
		Organizational culture and working climate	*"The dominant mindset of EMS centers is different. For example, innovation is encouraged in some places, but in many other places, people provide training only to fulfill certain requirements and the training is just a show."* (L21, male, 58yrs, training department leader, east region, provincial capital city)
	Leadership/champion	EMS center leadership	*"As the main leaders of our EMS centers, including those in charge of the training departments, we understand our current national level of training is less than 1%. This is a long-term need for us to improve and continue to promote."* (L6, female, 43yrs, training department leader, east region, provincial capital city)<br><br>*"Our leaders have changed frequently, and some leaders may not focus in public training so much."* (L19, female, 53yrs, training department leader, west region, provincial capital city)
		Training department leaders	*"I think the most important thing is the awareness and responsibility of the people involved in this work. If you look at the places where we know training is done well, their training leaders are all role models that we have been learning from."* (D9, male, 49yrs, EMS center deputy director, west region, provincial capital city)

		and program champions	*"Most people do training to get a job done. When you just make it a job, that's it. But work and career are two different things."* (D10, male, 59yrs, EMS center director, east region, prefecture-level city)
	Organizational staffing	BLS trainers' number and staff turnover	*"We are far from having enough trainers. We have only two full-time trainers in our department, and it's impossible for us two to train 10 million people in the city."* (L2, female, 46yrs, training department leader, central region, provincial capital city)<br><br>*"The problem is that we don't have full-time trainer, our trainers all have work and daily duty to do, so I can only arrange trainers who have a rest to conduct training, and they get no payment for that. I think this is the main barrier for us."* (D1, female, 47yrs, EMS center deputy director, west region, provincial capital city)
		Personnel regime and incentive measures	*"In fact, we have also encountered obstacles in recent years. For example, our personnel regime system, we are a full public welfare unit, we are not allowed to pay extra fees to our trainers, which will definitely hinder the future development."* (L18, male, 44yrs, training department leader, east region, centrally-administered municipality)<br><br>*"The loss of training personnel, to be honest, will also affect the development of the department."* (L14, female, 53yrs, training department leader, west region, centrally-administered municipality)
	Individual characteristics	BLS trainers' individual characteristics	*"The first promoting factor is that trainers of our center are all professionals, including doctors and specialists. Their initial motivation is to save people."* (L6, female, 43yrs, training department leader, east region, provincial capital city)<br><br>*"Most people living in our region are ethnic minorities, and many people still can't understand Mandarin, so we must bring some trainers who can speak dialects. They talk better, people listen better."* (T5, male, 50yrs, senior trainer, west region, provincial capital city)
		Experience in clinical, training, and certification	*"The trainers need to have rich clinical experience, or they may find it difficult to train laypeople well, or they don't have much cases to talk about. I think the experience is the key."* (D6, male, 39yrs, EMS center deputy director, central region, provincial capital city)<br><br>*"We have a national EMS skills competition, including a teaching competition. Many participates will become training champion because of these experiences."* (L23, male, 40yrs, training department leader, west region, provincial capital city)

		Recognition and sense of responsibility	*"I think this job absolutely needs a strong sense of responsibility and love! It is also true that we are all front-line EMS personnel, we see emergency situations, and we deeply understand its significance."* (T3, male, 35yrs, senior trainer, east region, provincial capital city)
	Quality & Fidelity monitoring	Quality control and improvement measures	*"For the quality control, we set standardized process to guide trainers, so that even the worst trainer can get the job done at an acceptable level."* (L11, male, 46yrs, training department leader, central region, provincial capital city)<br><br>*"We have issued a training requirement in accordance with the regional legislation, which is published on the official website. We have requirements for the venue, the ratio of trainers and trainees, including the ratio of trainees and manikins, in order to ensure its quality."* (L18, male, 44yrs, training department leader, east region, centrally-administered municipality)
		Enhancement of smart technologies	*"We have developed standardized courses, usually the training classes will be recorded, sometimes live broadcast."* (D10, male, 59yrs, EMS center director, east region, prefecture-level city)<br><br>*"We use public Wechat platforms and APPs a lot. We use smart phones to collect students' comments on courses and give feedback to trainers regularly."* (D11, male, 43yrs, EMS center deputy director, east region, provincial capital city)
		The authenticity of quality monitoring	*"We do annual assessment for our training sites, and we change the assessment method year by year. If we use fixed indicators, we might not find out what really happened."* (L14, female, 53yrs, training department leader, west region, centrally-administered municipality)
**Innovation factors**	Innovation fit	Training content fit with local needs	*"For BLS training for laypeople, less theory, more practice, use local language and get feedback. Our team also often goes to meetings to study how to make our classes more understandable and interesting to the public."* (L20, male, 50yrs, training department leader, west region, provincial capital city)
		Training output fit with EMS system	*"Nationally, the transformation of training is poor, trained public is still afraid to do CPR, and they don't know where someone needs help. We are gratified that our volunteer platform is connected with MPDS, and we have some volunteers who are very active."* (L24, male, 55yrs, training department leader, east region, provincial capital city)
		Training management fit	*"We used to use international standard courses before, but we found it cost too much. Moreover, the teaching methods and languages are somewhat different from those of our country, so we tried to*

		with administrative system	*make our own courses."* (D11, male, 43yrs, EMS center deputy director, east region, provincial capital city)
	Innovation characteristic s	The availability of qualified BLS courses	*"There is no uniform model of BLS training nationwide, and no uniform certification of courses, which I think is a hindrance factor, but also a reason for regional differences."* (L23, male, 40yrs, training department leader, west region, provincial capital city)
		The complexity and flexibility of BLS courses	*"According to our standard curriculum setting, the requirements for training personnel are quite high, and sometimes a class needs to use a whole department of our trainers, then the frequency of classes will not be too much, and the amount of training will be less."* (L5, male, 44yrs, training department leader, east region, centrally-administered municipality)
		Cost-effectiveness considerations	*"What our center requires is that trainees must be able to use the BLS skill after learning, so we try to reduce the number of trainees in each class. But after all training has a cost, and we cannot be charged, it will make our trainers feel that their work is not valuable."* (L6, female, 43yrs, training department leader, east region, provincial capital city)
	Innovation developer	The authority and reputation of EMS center	*"Our experience is that as long as the students have received our training, they should leave the impression of professional authority. They get something for themselves, and then they pass it on. Our brand is slowly standing up, and more and more people will be trained."* (L22, female, 58yrs, training department leader, west region, provincial capital city)
		The accreditation and qualification to issue certificates	*"The certification we issue to trainees are not recognized by other regions. I wonder if the national health commission can issue unified training and assessment standards and requirements. I think it's very necessary!"* (D8, male, 50yrs, EMS center deputy director, west region, provincial capital city)
**Bridging factors**	Community academic partnerships	Academy and associations of emergency medicine	*"The national emergency medicine association are doing work such as holding national conference and providing platforms for internal communications. We hope that they can make better specifications, things like national unified certification and so on."* (L23, male, 40yrs, training department leader, west region, provincial capital city)
		Cooperation with the education	*"We have worked hard here in the education system for many years, like we are a clinical training base for several universities. There are also first aid content written into the high school textbook."* (L14, female, 53yrs, training department leader, west region, centrally-administered municipality)

		system and researchers	*"I always think that what we lack is high-quality research and people with comprehensive ability in this area."* (D10, male, 59yrs, EMS center director, east region, prefecture-level city)
	Publicity and promotion	Publicity and promotion	*"I think there is another important problem that people are not aware of right now, and that is the publicity. In fact, publicity and training are two different things, publicity is to tell people that this thing is important, you must learn, training is to teach him how to do. But now the government is paying more attention to training, such as how many people I have trained this year. But you probably don't see it very often, for example, on any bus there's a TV screen that says how to find an AED or what an AED does. Right? Almost nothing."* (D11, male, 43yrs, EMS center deputy director, east region, provincial capital city)
	Purveyors/ intermediaries	Involvement of social organizations	*"Our local volunteer corps actually do a lot of work, they organize training and they have a good publicity effect."* (L24, male, 55yrs, training department leader, east region, provincial capital city)
		Support from manufacturers and technology companies	*"Some manikin factors are also working on training and related researches."* (L10, male, 48yrs, training department leader, central region, provincial capital city)<br>*"Our local AED companies have also actively participated in training program. They provided us with the AED model for free, and installed free AED in all the subways, which is very good, and actually saved people."* (T2, female, 61yrs, senior trainer, east region, provincial capital city)
